# Supplementary material for: Analysis of PPARγ Signaling Activity in Psoriasis
Source: Int J Mol Sci. 2021 Aug 10;22(16):8603. doi: 10.3390/ijms22168603 (PMC8395241; doi:10.3390/ijms22168603)
Supplement: Supplementary file 1 [file ijms-22-08603-s001.zip › Supplemental materials_Analysis of PPARg signaling activity in psoriasis/Pathway models/Models images and html files/Model of PPARG signaling in psoriasis/48382.html]

CLEC7A


# Protein CLEC7A

|  |  |
| --- | --- |
| URN | urn:agi-llid:64581 |
| Total Entities | 0 |
| Connectivity | 1145 |
| Name | CLEC7A |
| Description | C-type lectin domain family 7 member A |
| Notes | This gene encodes a member of the C-type lectin/C-type lectin-like domain (CTL/CTLD) superfamily. The encoded glycoprotein is a small type II membrane receptor with an extracellular C-type lectin-like domain fold and a cytoplasmic domain with an immunoreceptor tyrosine-based activation motif. It functions as a pattern-recognition receptor that recognizes a variety of beta-1,3-linked and beta-1,6-linked glucans from fungi and plants, and in this way plays a role in innate immune response. Alternate transcriptional splice variants, encoding different isoforms, have been characterized. This gene is closely linked to other CTL/CTLD superfamily members on chromosome 12p13 in the natural killer gene complex region. [provided by RefSeq, Jul 2008] |
| Primary Cell Localization | Plasma membrane |
| Class | Receptor |

---

|  |  |
| --- | --- |
| Pathway | Mannose Receptor Signaling |
|  | Dectin-1 (CLEC7A) Signaling |
|  | Antigen-Presenting Cell Role in Asthma |
|  | Proteins/Ions Transporters -> Expression Targets in Lymphoid System and Blood |
|  | pprarg neg, uknown targets, ps-positive |
|  | Model of PPARG signaling in psoriasis |
|  | 1\_Th2 cell response in asthma |
|  | 4\_2\_Genes with mutations currently associated with ulcerative colitis |
|  | 4\_1\_Polymorphisms associated with inflammatory bowel diseases |

---

|  |  |
| --- | --- |
| MedScan ID | 64581 |

---

|  |  |
| --- | --- |
| LocusLink ID | 64581 |
|  | 56644 |
|  | 502902 |

---

|  |  |
| --- | --- |
| Alias | dendritic cell-associated C-type lectin 1 |
|  | Dendritic cell-associated C-type lectin-1 beta |
|  | beta-glucan receptors |
|  | C-type (calcium dependent, carbohydrate recognition domain) lectin, superfamily member 12 |
|  | UNQ539/PRO1082 |
|  | SCARE2 |
|  | Clec7b |
|  | DECTIN-1 receptor |
|  | transmembrane protein dectin I |
|  | lectin-like receptor 1 |
|  | DC-associated C-type lectin 1 |
|  | CD369 |
|  | RGD1565140 |
|  | c-type lectin domain family 7, member a |
|  | DECTIN I |
|  | lectin-like receptor I |
|  | DECTIN-1 receptors |
|  | Clecsf12 |
|  | clec7a |
|  | hDectin I |
|  | DECTIN1 |
|  | beta-glucan receptor |
|  | hDectin-1 |
|  | C-type lectin, superfamily member 12 |
|  | transmembrane protein dectin-1 |
|  | C-type lectin, superfamily member XII |
|  | CANDF4 |
|  | Beta-glucan receptor isoform B |
|  | C-type lectin domain containing 7A |
|  | Lectin-like receptor 1B |
|  | beta-gr |
|  | BGR |
|  | C-type (calcium dependent, carbohydrate-recognition domain) lectin, superfamily member 12 |
|  | C-type lectin superfamily member 12 |
|  | dectin-1 |
|  | dendritic cell-associated C-type lectin-1 |
|  | C-type lectin domain family 7 member A |

---

|  |  |
| --- | --- |
| GO ID | 0001872 |
|  | 0042287 |
|  | 0030246 |
|  | 0046872 |
|  | 0008329 |
|  | 0042110 |
|  | 0009756 |
|  | 0008037 |
|  | 0071226 |
|  | 0042832 |
|  | 0016046 |
|  | 0032491 |
|  | 0006954 |
|  | 0045087 |
|  | 0002221 |
|  | 0006910 |
|  | 1903431 |
|  | 0030335 |
|  | 0008284 |
|  | 0043280 |
|  | 1900017 |
|  | 0010628 |
|  | 0045078 |
|  | 0032731 |
|  | 0045082 |
|  | 0045084 |
|  | 0045086 |
|  | 0045410 |
|  | 0045416 |
|  | 0051712 |
|  | 0051251 |
|  | 0071639 |
|  | 0045429 |
|  | 0050766 |
|  | 0031334 |
|  | 0060267 |
|  | 0032930 |
|  | 0042535 |
|  | 0090303 |
|  | 0009986 |
|  | 0005737 |
|  | 0016021 |
|  | 0043231 |
|  | 0005654 |
|  | 0005886 |
|  | 0002366 |
|  | 0002223 |
|  | 0001874 |
|  | 0001846 |
|  | 0030247 |
|  | 0002752 |
|  | 0001879 |
|  | 0002376 |
|  | 0006911 |
|  | 0032760 |
|  | 0002238 |
|  | 0001878 |
|  | 0016337 |
|  | 0009897 |
|  | 0016020 |

---

|  |  |
| --- | --- |
| KEGG ID | hsa:64581 |

---

|  |  |
| --- | --- |
| Organism | Homo sapiens {Organism urn:agi-taxid:9606} |
|  | Mus musculus {Organism urn:agi-taxid:10090} |
|  | Rattus norvegicus {Organism urn:agi-taxid:10116} |
|  | Homo sapiens |
|  | Mus musculus |
|  | Rattus norvegicus |

---

|  |  |
| --- | --- |
| Mouse chromosome position | 6 |

---

|  |  |
| --- | --- |
| OMIM ID | 606264 |
|  | 614079 |
|  | 613108 |

---

|  |  |
| --- | --- |
| Rat chromosome position | 4q42 |

---

|  |  |
| --- | --- |
| Hugo ID | 14558 |
|  | HGNC:14558 |

---

|  |  |
| --- | --- |
| Human chromosome position | 12p13.2 |

---

|  |  |
| --- | --- |
| Swiss-Prot Accession | Q68D78 |
|  | Q9BXN2 |
|  | A0A024RAN9 |
|  | A0A0S2Z5Q1 |
|  | Q9BXN2.1 |
|  | V9GXI5 |
|  | Q6QLQ4.3 |
|  | B2RYG9 |
|  | B5BUZ1 |
|  | B5BUZ2 |
|  | B2R861 |
|  | B7Z494 |
|  | B7Z5A9 |
|  | B7Z5B9 |
|  | Q6IPS7 |
|  | Q96D32 |
|  | Q96DR9 |
|  | Q96LD3 |
|  | Q96PA4 |
|  | Q96PA5 |
|  | Q96PA6 |
|  | Q96PA7 |
|  | Q96PA8 |
|  | Q9H1K3 |
|  | Q6QLQ4 |

---

|  |  |
| --- | --- |
| GenBank ID | NC\_000012 |
|  | NR\_125336 |
|  | NM\_197947 |
|  | NP\_922938 |
|  | NM\_197950 |
|  | NP\_922941 |
|  | NM\_197949 |
|  | NP\_922940 |
|  | NM\_022570 |
|  | NP\_072092 |
|  | NM\_197948 |
|  | NP\_922939 |
|  | XM\_017019822 |
|  | XP\_016875311 |
|  | XM\_017019823 |
|  | XP\_016875312 |
|  | XM\_006719135 |
|  | XP\_006719198 |
|  | XM\_024449133 |
|  | XP\_024304901 |
|  | XM\_024449132 |
|  | XP\_024304900 |
|  | NM\_197954 |
|  | NP\_922945 |
|  | NG\_016291 |
|  | AC024224 |
|  | CH471094 |
|  | EAW96144 |
|  | EAW96145 |
|  | EAW96146 |
|  | EAW96147 |
|  | EAW96148 |
|  | EAW96149 |
|  | EAW96150 |
|  | EAW96151 |
|  | EAW96152 |
|  | EAW96153 |
|  | EAW96154 |
|  | EAW96155 |
|  | CQ779237 |
|  | CAF63003 |
|  | CQ779239 |
|  | CAF63006 |
|  | AA361063 |
|  | AF313468 |
|  | AAK37473 |
|  | AF313469 |
|  | AAK37474 |
|  | AF400595 |
|  | AAL11711 |
|  | AF400596 |
|  | AAL11712 |
|  | AF400597 |
|  | AAL11713 |
|  | AF400598 |
|  | AAL11714 |
|  | AF400599 |
|  | AAL11715 |
|  | AF400600 |
|  | AAL11716 |
|  | AF400601 |
|  | AAL11717 |
|  | AF400602 |
|  | AAL11718 |
|  | AJ312372 |
|  | CAC43846 |
|  | AJ312373 |
|  | CAC43847 |
|  | AK294111 |
|  | BAH11674 |
|  | AK295108 |
|  | BAH11976 |
|  | AK297028 |
|  | BAH12480 |
|  | AK298679 |
|  | BAH12845 |
|  | AK298724 |
|  | BAH12855 |
|  | AK313247 |
|  | BAG36058 |
|  | AY009090 |
|  | AAG33923 |
|  | AY026769 |
|  | AAK20114 |
|  | AY026770 |
|  | AAK20115 |
|  | AY026771 |
|  | AAK20116 |
|  | AY358685 |
|  | AAQ89048 |
|  | AY359002 |
|  | AAQ89361 |
|  | BC013385 |
|  | AAH13385 |
|  | BC071746 |
|  | AAH71746 |
|  | BC093829 |
|  | AAH93829 |
|  | BC093831 |
|  | AAH93831 |
|  | CR749536 |
|  | CAH18342 |
|  | DA193873 |
|  | KU178745 |
|  | ALQ34203 |
|  | KU178746 |
|  | ALQ34204 |
|  | LC369590 |
|  | BBC70867 |
|  | LC369591 |
|  | BBC70868 |
|  | Q9BXN2 |
|  | NC\_000072 |
|  | NM\_001309637 |
|  | NP\_001296566 |
|  | NR\_132130 |
|  | NM\_020008 |
|  | AC138620 |
|  | CH466523 |
|  | EDK99926 |
|  | EDK99927 |
|  | CQ779241 |
|  | CAF63010 |
|  | AA240643 |
|  | AF262985 |
|  | AAF72710 |
|  | AY534909 |
|  | AAS37670 |
|  | BC027742 |
|  | AAH27742 |
|  | BY547544 |
|  | BY750022 |
|  | Q6QLQ4 |
|  | NC\_005103 |
|  | XM\_006237100 |
|  | XP\_006237162 |
|  | NM\_001173386 |
|  | NP\_001166857 |
|  | XR\_001837491 |
|  | AC\_000072 |
|  | AABR07073062 |
|  | AAHX01033063 |
|  | CH473964 |
|  | EDM01739 |
|  | EDM01740 |
|  | AB455106 |
|  | BAG69160 |
|  | AB455107 |
|  | BAG69161 |
|  | BC166774 |
|  | AAI66774 |
|  | GU357485 |
|  | ADK94895 |
|  | XM\_005253467 |
|  | XP\_005253524 |
|  | NC\_018923 |
|  | AMYH02026022 |
|  | HQ447540 |
|  | ADQ32026 |
|  | NP\_064392 |
|  | AC\_000028 |
|  | AAHY01057557 |
|  | AAHY01057558 |
|  | AAHY01057559 |
|  | XM\_006719136 |
|  | XP\_006719199 |
|  | XM\_005253468 |
|  | XP\_005253525 |
|  | AC\_000144 |
|  | ABBA01067796 |

---

|  |  |
| --- | --- |
| Swiss-Prot ID | CLC7A\_HUMAN |

---

|  |  |
| --- | --- |
| Cell Localization | Cell membrane |
|  | Cytoplasm |

---

|  |  |
| --- | --- |
| Ensembl ID | ENSG00000172243 |
|  | ENSP00000266456.6 |
|  | ENST00000353231.9 |
|  | ENSP00000302569.8 |
|  | ENST00000304084.12 |
|  | ENSP00000431461.1 |
|  | ENST00000533022.5 |
|  | ENSP00000298523.5 |
|  | ENST00000298523.9 |
|  | ENSP00000379743.2 |
|  | ENST00000396484.6 |
|  | ENSP00000312089.4 |
|  | ENST00000310002.4 |
|  | ENSP00000434392.1 |
|  | ENST00000531192.5 |
|  | ENSMUSG00000079293 |
|  | ENSMUSP00000139167.1 |
|  | ENSMUST00000184581.2 |
|  | ENSMUST00000195589.5 |
|  | ENSRNOG00000054251 |
|  | ENSRNOP00000073639.1 |
|  | ENSRNOT00000091552.1 |
|  | ENSRNOP00000069723.1 |
|  | ENSRNOT00000077793.1 |
|  | ENSP00000266456 |
|  | ENST00000353231 |
|  | ENSP00000302569 |
|  | ENST00000304084 |
|  | ENSP00000431461 |
|  | ENST00000533022 |
|  | ENSP00000298523 |
|  | ENST00000298523 |
|  | ENSP00000379743 |
|  | ENST00000396484 |
|  | ENSP00000312089 |
|  | ENST00000310002 |
|  | ENSP00000432876 |
|  | ENST00000529761 |
|  | ENSP00000434392 |
|  | ENST00000531192 |
|  | ENSMUSP00000141234 |
|  | ENSMUST00000195589 |
|  | ENSRNOP00000073639 |
|  | ENSRNOT00000091552 |
|  | ENSRNOP00000069723 |
|  | ENSRNOT00000077793 |
|  | ENSRNOG00000033879 |
|  | ENSRNOP00000056630 |
|  | ENSRNOT00000059885 |

---

|  |  |
| --- | --- |
| MGI ID | MGI:1861431 |

---

|  |  |
| --- | --- |
| RGD ID | 1565140 |

---

|  |  |
| --- | --- |
| Unigene ID | Hs.143929 |
|  | Mm.239516 |
|  | Rn.15743 |

---

|  |  |
| --- | --- |
| Homologene ID | 49606 |

---

|  |  |
| --- | --- |
| Shape | Stick-vertex |

---
